# Antipsychotic treatment patterns in refugees and their Swedish-born peers with first-episode non-affective psychosis: findings from the REMAIN study

**DOI:** 10.1192/bjo.2023.38

**Published:** 2023-04-04

**Authors:** Julia Spaton Goppers, Ellenor Mittendorfer-Rutz, Alexis E. Cullen, Christopher Jamil de Montgomery, Antti Tanskanen, Marie Norredam, Heidi Taipale

**Affiliations:** Department of Clinical Neuroscience, Division of Insurance Medicine, Karolinska Institutet, Sweden; Department of Clinical Neuroscience, Division of Insurance Medicine, Karolinska Institutet, Sweden; and Department of Psychosis Studies, Institute of Psychiatry, Psychology and Neuroscience, King's College London, UK; Department of Clinical Neuroscience, Division of Insurance Medicine, Karolinska Institutet, Sweden; and Department of Public Health, Danish Research Centre for Migration, Ethnicity and Health (MESU), University of Copenhagen, Denmark; Department of Clinical Neuroscience, Division of Insurance Medicine, Karolinska Institutet, Sweden; and Department of Forensic Psychiatry, University of Eastern Finland, Niuvanniemi Hospital, Finland; Department of Public Health, Danish Research Centre for Migration, Ethnicity and Health (MESU), University of Copenhagen, Denmark

**Keywords:** Psychotic disorders, refugees, antipsychotics, epidemiology, schizophrenia

## Abstract

**Background:**

Previous studies suggest that migrants tend to utilise antipsychotics less often than their native-born peers. However, studies examining antipsychotic use among refugees with psychosis are lacking.

**Aims:**

To compare the prevalence of antipsychotic drug use during the first 5 years of illness among refugees and Swedish-born individuals with a newly diagnosed non-affective psychotic disorder, and to identify sociodemographic and clinical factors associated with antipsychotic use.

**Method:**

The study population included refugees (*n* = 1656) and Swedish-born persons (*n* = 8908) aged 18–35 years during 2007–2018, with incident diagnosis of non-affective psychotic disorder recorded in the Swedish in-patient or specialised out-patient care register. Two-week point prevalence of antipsychotics use was assessed every 6 months in the 5 years following first diagnosis. Factors associated with antipsychotic use (versus non-use) at 1 year after diagnosis were examined with modified Poisson regression.

**Results:**

Refugees were somewhat less likely to use antipsychotics at 1 year after first diagnosis compared with Swedish-born persons (37.1% *v*. 42.2%, age- and gender-adjusted risk ratio 0.88, 95% CI 0.82–0.95). However, at the 5-year follow-up, refugees and Swedish-born individuals showed similar patterns of antipsychotic use (41.1% *v*. 40.4%). Among refugees, higher educational level (>12 years), previous antidepressant use and being diagnosed with schizophrenia/schizoaffective disorder at baseline were associated with an increased risk of antipsychotics use, whereas being born in Afghanistan or Iraq (compared with former Yugoslavia) was associated with decreased risk.

**Conclusions:**

Our findings suggest that refugees with non-affective psychotic disorders may need targeted interventions to ensure antipsychotic use during the early phase of illness.

Non-affective psychotic disorders such as schizophrenia can be lifelong conditions that significantly affect an individual's ability to maintain social relationships and secure gainful employment.^1^ Antipsychotic medication is an essential component of effective treatment, and has been shown to reduce symptom severity and risk of relapse.^2^ Indeed, evidence suggests that individuals who commence antipsychotic treatment early in the course of illness are more likely to achieve recovery compared with those who start treatment later and those who never receive treatment.^3^ The optimal duration of antipsychotic treatment varies across diagnostic groups: lifelong treatment is recommended for chronic schizophrenia and schizoaffective disorder, whereas recommended duration of antipsychotic treatment for other non-affective psychotic disorders is less clear.^2,3^

Despite ample evidence emphasising the importance of antipsychotic treatment, many persons with psychotic disorders are either unwilling to use them or discontinue their treatment, potentially because of side-effects^4^ and/or poor insight.^5^ Previous research has shown that antipsychotic discontinuation is associated with low socioeconomic status, younger age, poor health literacy, substance use disorders, negative attitudes toward medication, being part of an ethnic minority group and experiencing barriers to healthcare.^5,6^ One of the main strategies for improving medication adherence is the use of long-acting injectable drugs (LAIs).^7^ Previous studies have shown that LAIs reduce the risk of hospital readmission by 20–30% compared with oral medications.^8^ Compared with other oral antipsychotics, clozapine has been associated with reduced risk of hospital readmission and all-cause mortality.^9^ This was further supported by a recent meta-analysis which showed that LAIs and clozapine were associated with a significant reduction in all-cause mortality in patients with schizophrenia,^10^ where clozapine contributed to a particularly large protective effect, especially in terms of suicide-related deaths.^10^

## Refugees and psychosis

There is consistent evidence indicating that refugees are at increased risk of developing non-affective psychotic disorders such as schizophrenia.^11^ This may be because of increased exposure to traumatising events (e.g. war, conflict and loss/lack of contact with family members), the social and financial challenges of leaving one's home abruptly, and post-migration stressors (e.g. acculturation stress, social isolation and experiences of discrimination).^12^ Despite experiencing an elevated risk of developing mental disorders, refugees are less likely to utilise psychiatric healthcare services and to use antipsychotic and other psychopharmacotherapies than individuals in their host country.^13–15^ A recent study from Finland indicated that this is particularly the case for migrants from Asia and Sub-Saharan Africa, who were found to be less likely to purchase psychotropic drugs compared with migrants from Western countries.^16^ Several factors have been hypothesised to explain these findings, including difficulties navigating healthcare systems (potentially because of social or language barriers) and stigma associated with mental disorders.^17,18^ However, to our knowledge, there have been no previous studies investigating the extent to which demographic and clinical factors are associated with antipsychotic use in refugee populations.

Despite the known treatment gaps for refugees, it is unclear whether refugees with newly diagnosed psychotic disorders differ in terms of prevalence of antipsychotic use or LAI initiation from their native-born peers in their host countries. Previous studies have typically assessed the frequency of antipsychotics in migrants in general, with few studies examining refugees specifically^19^ or their patterns of antipsychotic use after first diagnosis.^20^ As noted above, little is known about the factors associated with antipsychotic use among refugees, and identifying these factors may enable targeted interventions to promote antipsychotic use among young refugees with non-affective psychotic disorders during the early stages of illness. To this end, we aimed to compare pharmacological treatment patterns in young refugees with first-episode non-affective psychotic disorders and their Swedish-born peers. We also aimed to identify sociodemographic and clinical factors associated with antipsychotic use in refugees and their Swedish-born peers at 1 year after diagnosis, and compare time to LAI or clozapine initiation in these populations.

## Method

### Study design

We conducted a nationwide, register-based cohort study. As there have been previous studies examining antipsychotic use among migrants^15,16,19^ but there is an absence of studies focusing on refugees specifically, this study was restricted to comparisons between refugee youth and Swedish-born youth (i.e. non-refugee migrants were not included). The study population comprised refugees and Swedish-born persons aged 18–35 years, residing in Sweden during 2007–2018, with incident diagnosis of non-affective psychotic disorder according to the ICD-10 (codes F20–F29), in either in-patient or specialised out-patient care. The incident cases were defined as being a resident in Sweden for a minimum of three calendar years before the first diagnosis and without a recorded diagnosis of non-affective psychosis during that period. A 1-year washout period for antipsychotic use was applied to maximise the likelihood of capturing incident cases. Individuals who had been dispensed antipsychotics between 3 and 15 months before the first diagnosis were excluded from the study. Initiation of antipsychotic treatment 0–3 months before first diagnosis was allowed so as not to exclude people who first presented to primary care services before entering specialised healthcare services. The final study population comprised 1656 refugees and 8908 Swedish-born individuals.

The pseudonymised data used in this study were obtained from nationwide Swedish registers and linked by personal identification numbers that had been assigned to all residents at birth or at immigration. The study participants were identified from the Swedish National Patient Register (NPR), kept by the Board of Health and Welfare, covering in-patient and specialised out-patient healthcare visits. The NPR was used to determine patient diagnosis at first contact and hospital admissions occurring in the 3 years before first diagnosis and up to end of follow-up. Information on dispensing of prescribed medications was obtained from the Prescribed Drug Register (PDR), with information on dates and causes of death obtained from the Cause of Death Register (both registers maintained by the Board of Health and Welfare). Two registers held by Statistics Sweden, the Longitudinal Database for Health Insurance and Labour Market Studies (LISA) and the Longitudinal Database for Integration Studies (STATIV), were used to obtain data on demographic factors, information on work disability and unemployment, and refugee status.

### Exposure assessment

The primary exposure variable was refugee status, categorised as refugee versus Swedish-born person. A refugee was defined as a person who has been granted a residence permit in Sweden who had fled war, violence, conflict or persecution and who had crossed an international border to find safety in another country. Additionally, family reunifications to refugees were included as these persons were assumed to have similar background to as refugees. Swedish-born individuals were defined as persons born in Sweden with at least one parent born in Sweden.

### Outcome assessment

The primary outcome of this study was 2-week point prevalence of antipsychotic use, assessed every 6 months from diagnosis until the end of follow-up with a maximum of 5 years. The outcome for the regression models was the prevalence of antipsychotic (use versus non-use) at 1 year after first treatment for psychosis. For the subanalyses of LAI and clozapine initiation, the outcome was defined as the first dispensing of any LAI or clozapine, analysed in two separate models. The antipsychotics in this study were defined as Anatomical Therapeutic Chemical (ATC) code N05A, excluding lithium (ATC code N05AN01). Clozapine (ATC code N05AH02) and LAIs, defined through drug package information, were assessed separately.

**Fig. 1:**
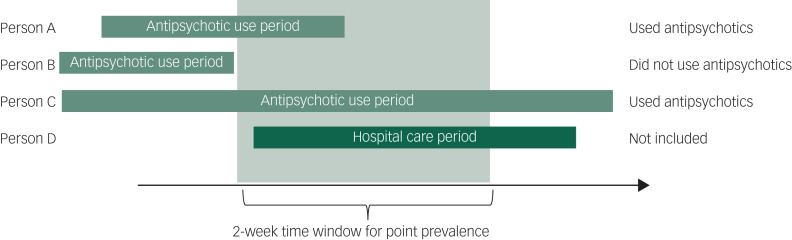
Definition of point prevalence of antipsychotic use in a 2-week time window. Person A used antipsychotics during the 2-week period (on at least 1 out of 14 days). Person B did not use antipsychotics during the 2-week period (may or may not have had antipsychotic use outside of this time window). Person C used antipsychotics during the 2-week period. Person D is not included in the prevalence calculation because they stayed in hospital care for ≥10 days out of the 2-week time period and their exposure status is therefore unknown.

The prevalence of antipsychotic use was assessed every 6 months, censoring for emigration, death and end of data linkage (31 December 2018). The prevalence was assessed as a 2-week point prevalence, as use versus non-use of antipsychotic drugs during the 2-week time window (Fig. 1). From each time point, individuals who were censored owing to death, emigration or end of data linkage, and those who stayed in hospital care for ≥10 days of the 2-week time period, did not contribute to the prevalence calculation. Thus, the sample size varied between the time points. Antipsychotic use was based on drug use periods, assigned by dates when drug use started and ended, generated with the PRE2DUP; a method based on mathematical modelling of drug dispensing data recorded in the PDR.^21^ In the PRE2DUP method, medicines dispensed from the pharmacy are processed in chronological order and by considering each individual's previous purchase history for every ATC code. Thus, the method enables construction of exposure time periods and estimates of the consumed dose during the period by taking the purchased amount in defined daily doses into account. Stockpiling of drugs, personal purchasing patterns such as continuity in purchases, and periods of in-patient care during which medication use was not registered were also considered.

### Covariates

We adjusted for somatic and mental conditions recorded in specialised healthcare based on the rationale that regular healthcare contact might increase adherence to antipsychotic medications, and because antipsychotics may be used for treatment of other conditions or side-effects associated with treatments of certain conditions (e.g. cancer). Supplementary Table 1 available at https://doi.org/10.1192/bjo.2023.38 describes sociodemographic and clinical covariates used in this study, and Supplementary Table 2 provides details about the categorisation of the countries of birth. The sociodemographic covariates were measured at the time of first diagnosis or during the previous year. Marital status, education and living area were assessed on 31 December in the year before cohort entry, and unemployment and sickness absence were assessed as the number of days during the previous calendar year. Clinical covariates were measured during the 3 years before first diagnosis; other psychotropic medications were measured during the 3 months before diagnosis (Supplementary Table 1). First diagnosis was categorised as schizophrenia/schizoaffective disorder (ICD-10 codes F20, F25), acute/ transient psychosis (ICD-10 code F23), unspecified psychosis (ICD-10 code F29) or other psychosis (ICD-10 codes F21, F22, F24, F28).

### Data analysis

All statistical analyses were performed with the software Stata/IC version 16 for Windows. Individuals entered the cohort at the date of their first treatment contact for any non-affective psychotic disorder and were followed up for a maximum of 5 years. The point prevalence of antipsychotic use with 95% confidence intervals were calculated as proportions and were compared between refugees and Swedish-born persons. Factors associated with antipsychotic use (versus non-use) were assessed by fitting modified Poisson regression models with robust error variance estimation,^22^ using the point prevalence of antipsychotic use at 1 year as the outcome. The models were stratified by refugee status. The results are reported as risk ratios and their 95% confidence intervals.

Time to LAI initiation and time to clozapine initiation was examined in two separate Cox proportional regression models, and reported as hazard ratios with 95% confidence intervals. In these models, refugee status was used as an independent variable in both crude and adjusted models, in which the sociodemographic covariates also were included. Analyses were censored at death, emigration and end of data linkage (31 December 2018). Proportional hazard assumption was verified by log–log plots and on the basis of Schoenfeld residuals.

The data used in this study contained some missing values: educational level was missing for 366 observations (<3.5% of the sample), which were assigned to the lowest category (<10 years) because it can be assumed that individuals with missing data on education most likely belonged to that category.^23^ The marital status variable contained two missing values, and these cases were assigned to the unmarried category as this was most prevalent in the current sample.

### Ethics approval and consent to participate

The research project was approved by the Regional Ethical Board of Stockholm (dnr 2007/762-31; 2016/1533-32).The authors assert that all procedures contributing to this work comply with the ethical standards of the relevant national and institutional committees on human experimentation and with the Helsinki Declaration of 1975, as revised in 2008. No experiments were done with humans or animals, and the study utilised data gathered in administrative registers. Informed consent was not sought because of the registry-based nature of the study where data is pseudonymised, so no specific individual could be identified.

## Results

Men formed the majority of the study population, comprising 72% of the refugees and 65% of the Swedish-born individuals (Table 1). The mean age of first contact for non-affective psychotic disorders (i.e. time of first diagnosis) was 26.2 years for the refugees and 25.8 years for Swedish-born persons. The mean age at immigration for the refugees was 12.5 years; 59.4% of refugees had formally resided in Sweden for >10 years before their first diagnosis. Approximately half (50.5%) of the refugees had a low educational level (<10 years), and 36.4% had attained a higher level (>12 years). Among the Swedish-born people, more than half (56.5%) had higher education. There was higher prevalence of unemployment in refugees compared with Swedish-born persons; however, refugees were slightly less likely to have received sickness absence payments and disability pension than their native-born peers. Substance use disorders and common mental disorders were more prevalent in the Swedish-born population than in refugees, whereas attention-deficit hyperactivity disorder was far more prevalent among Swedish-born individuals. Swedish-born individuals were also more likely to have received treatment for suicide attempts in the 3 years before first diagnosis of non-affective psychotic disorder, and were more likely to have used other psychotropic drugs.

**Table 1:** Descriptive statistics of baseline sociodemographic characteristics of refugees and Swedish-born individuals with non-affective psychosis

Baseline characteristics	Refugees, *n* (%), *n* = 1656 (15.7%)	Swedish-born individuals, *n* (%), *n* = 8908 (84.3%)	Total, *n* (%), *N* = 10 564 (100%)
Female	464 (28.0)	3121 (35.0)	3585 (33.9)
Male	1192 (72.0)	5787 (65.0)	6979 (66.1)
Age at first diagnosis, years, mean±s.d.	26.2 (4.8)	25.8 (4.9)	25.9 (4.9)
Age categories, years			
18–23	579 (35.0)	3309 (37.2)	3888 (36.8)
24–29	608 (36.7)	3275 (36.8)	3883 (36.8)
30–35	469 (28.3)	2324 (26.1)	2793 (26.4)
Duration of residency in Sweden before diagnosis			
3–5 years	319 (19.3)		
6–10 years	353 (21.3)		
>10 years	984 (59.4)		
Age at the year of immigration, years, mean±s.d.	12.5 (6.7)		
Educational level			
Low (<10 years)	836 (50.5)	3208 (36.0)	4044 (38.3)
Medium (10–12 years)	217 (13.1)	821 (9.2)	1038 (10.2)
High (>12 years)	603 (36.4)	4879 (56.5)	5482 (54.8)
Type of living area			
Cities (densely populated)	902 (54.5)	3691 (41.4)	4593 (43.5)
Towns and suburbs	593 (35.8)	3572 (40.1)	4165 (39.4)
Rural areas (thinly populated)	161 (9.7)	1645 (18.5)	1806 (17.1)
Unemployment			
No unemployment	917 (55.4)	6333 (71.1)	7250 (68.6)
1–180 days	564 (34.1)	2084 (23.4)	2648 (25.1)
>180 days	175 (10.6)	491 (5.5)	666 (6.3)
Sickness absence			
0 gross days	1504 (90.8)	7609 (85.4)	9113 (86.3)
1–90 gross days	85 (5.1)	732 (8.2)	817 (7.7)
>90 gross days	67 (4.1)	567 (6.4)	634 (6.0)
Disability pension			
No	1520 (91.8)	7693 (86.4)	9213 (87.2)
Yes	136 (8.2)	1215 (13.6)	1351 (12.8)
Marital status			
Unmarried	1270 (76.7)	8432 (94.7)	9702 (91.8)
Married	267 (16.1)	362 (4.1)	629 (6.0)
Divorced	119 (7.2)	114 (1.3)	233 (2.2)
Region of birth			
Afghanistan	86 (5.2)		
Iraq	309 (18.7)		
Iran	121 (7.3)		
Other Middle East	154 (9.3)		
Somalia	294 (17.8)		
Other Africa	185 (11.2)		
Former Yugoslavia	292 (17.6)		
Other Europe	103 (6.2)		
The Americas and Asia	112 (6.8)		
Type of psychotic disorder			
Schizophrenia (F20), schizoaffective disorders (F25)	165 (10)	787 (8.8)	952 (9.0)
Schizotypal disorder (F21), persistent delusional disorder (F22), induced delusional disorder (F24), other nonorganic psychotic disorders (F28)	165 (10)	1009 (11.3)	1174 (11.1)
Acute and transient psychotic disorders (F23)	562 (33.9)	3330 (37.4)	3892 (36.8)
Unspecified nonorganic psychosis (F29)	764 (46.1)	3782 (42.5)	4546 (43.0)
Clinical conditions/comorbidities			
Substance use disorders	269 (16.2)	1834 (20.6)	2103 (19.9)
Bipolar disorder	26 (1.6)	285 (3.20)	311 (2.9)
Personality disorder	43 (2.6)	410 (4.6)	453 (4.3)
Attention-deficit hyperactivity disorder	62 (3.7)	1014 (11.4)	1076 (10.2)
Common mental disorders	314 (19.0)	2505 (28.1)	2819 (26.7)
Musculoskeletal disease	158 (9.5)	779 (8.7)	937 (8.9)
Cardiovascular disease	34 (2.1)	172 (1.9)	206 (2.0)
Cancer	28 (1.7)	238 (2.7)	266 (2.5)
Previous suicide attempt	79 (4.8)	628 (7.1)	707 (6.7)
Previous medication use			
Antidepressant drugs	187 (11.3)	1827 (20.5)	2014 (19.1)
Anxiolytic or hypnotic drugs	254 (15.3)	2183 (24.5)	2437 (23.1)
Mood stabilisers	28 (1.7)	287 (3.2)	315 (3.0)

Detailed descriptions of the covariates and the country categorisation are enclosed as Supplementary Tables 1 and 2.

Across both groups, unspecified, non-organic psychosis and acute and transient psychotic disorders were the most common diagnoses. Among the refugees, 10.0% were diagnosed with schizophrenia/schizoaffective disorder at baseline compared with 8.8% of Swedish-born individuals.

### Point prevalence of antipsychotic use

**Fig. 2:**
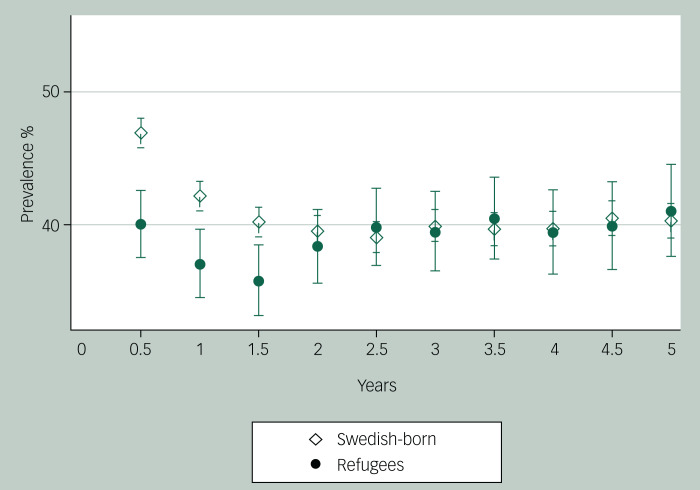
Point prevalence (%) of antipsychotic use with 95% confidence intervals for refugees and Swedish-born individuals from 6 months to 5 years after first diagnosis of non-affective psychotic disorder. Baseline *N* = 10 564, year 1 *N* = 9053, year 5 *N* = 5564.

At 1 year after diagnosis, the prevalence of any antipsychotic use was lower among refugees (37.1%, 95% CI 34.6–39.7) than in Swedish-born individuals (42.2%, 95% CI 41.1–43.3) (Fig. 2, Supplementary Table 3). Prevalence of use differed to a lesser extent at 2 years after the first diagnosis (38.4% *v*. 39.6%), but at 3 years after diagnosis the difference between the groups diminished (39.5% *v*. 40.0%). When 5 years had passed, the proportions converged such that the prevalence was similar among both refugees and Swedish-born persons (41.1% and 40.4%, respectively).

Among refugees, the birth country with the largest proportion of antipsychotic users at 1 year after diagnosis was former Yugoslavia (43.9%), followed by the other European countries (41.1%). Individuals born in Afghanistan were least likely to use antipsychotics.

Among those who were diagnosed with schizophrenia/schizoaffective disorder, 49% used antipsychotics at the 1-year follow-up, which was the largest proportion among all other diagnosis categories (data not shown).

### Factors associated with antipsychotic drug use

At 1 year after first diagnosis, refugees were 12% less likely to use antipsychotics relative to Swedish-born youth, after adjustment for age and gender (adjusted risk ratio 0.88, 95% CI 0.82–0.95). In the adjusted models stratified by refugee status, having attained a high educational level (>12 years) was associated with increased chances of antipsychotic use in both refugees and Swedish-born individuals (compared with <10 years), especially among refugees (adjusted risk ratio 1.30, 95% CI 1.10–1.53; Swedish-born individuals: adjusted risk ratio 1.09, 95% CI 1.02–1.16) (Table 2). Receiving disability pension at baseline was also associated with increased risk of using antipsychotics, but only among Swedish-born persons (adjusted risk ratio 1.30, 95% CI 1.21–1.40). Previous antidepressant use was associated with increased chances of antipsychotic use among refugees and Swedish-born persons. Those born in Afghanistan presented the lowest adjusted risk ratios among all other included regions, with individuals from this country being 51% less likely to use antipsychotics compared with those born in former Yugoslavia (adjusted risk ratio 0.49, 95% CI 0.32–0.76). All other regions included in this study similarly presented lower chances of antipsychotic use compared with former Yugoslavia; however, these associations (except for Iraq) were not statistically significant. Those diagnosed with schizophrenia/schizoaffective disorder at baseline were more likely to use antipsychotics compared with the reference group ‘other psychotic disorders’. The adjusted risk ratio of this baseline diagnosis type was 1.72 (95% CI 1.27–2.34) for refugees and 1.32 (95% CI 1.17–1.48) for Swedish-born persons.

**Table 2:** Unadjusted and adjusted modified Poisson regression models of the association between sociodemographic and clinical factors associated with antipsychotic use (versus non-use) at 1 year after diagnosis of non-affective psychosis in Sweden stratified by refugee status

	Refugees (*n* = 1377)		Swedish-born individuals (*n* = 7676)	
	Unadjusted risk ratio (95% CI)	Adjusted risk ratio (95% CI)^a^	Unadjusted odds ratio (95% CI)	Adjusted odds ratio (95% CI)^a^
Gender (reference male)	1.09 (0.94–1.26)	1.03 (0.88–1.19)	1.00 (0.95–1.06)	0.98 (0.93–1.04)
Age at first diagnosis (reference 18–23 years)				
24–29 years	1.02 (0.86–1.19)	0.97 (0.82–1.16)	1.04 (0.98–1.11)	1.02 (0.96–1.09)
30–35 years	0.99 (0.83–1.18)	1.01 (0.82–1.24)	1.00 (0.94–1.07)	1.01 (0.94–1.09)
Age at year of immigration		Not applicable (overlaps)	Not applicable	Not applicable
Duration of residency (reference 3–5 years)				
6–10 years	1.09 (0.87–1.35)	1.04 (0.84–1.30)	Not applicable	Not applicable
>10 years	1.01 (0.84–1.22)	0.83 (0.67–1.02)		
Living area (reference dense cities)				
Towns and suburbs	1.15 (1.00–1.33)	1.14 (0.98–1.32)	0.97 (0.92–1.03)	0.98 (0.93–1.04)
Rural areas	1.03 (0.81–1.33)	1.00 (0.78–1.29)	0.98 (0.91–1.05)	1.01 (0.94–1.09)
Education level (reference low, <10 years)				
Medium (10–12 years)	0.99 (0.79–1.25)	1.05 (0.83–1.33)	0.92 (0.83–1.03)	0.92 (0.83–1.03)
High (>12 years)	1.28 (1.11–1.47)	1.30 (1.10–1.53)^b^	1.08 (1.02–1.15)	1.09 (1.02–1.16)
Unemployment (reference no unemployment)				
1–180 days	0.86 (0.73–1.00)	0.88 (0.75–1.03)	0.94 (0.89–1.01)	0.99 (0.93–1.06)
>180 days	0.88 (0.70–1.12)	0.93 (0.73–1.17)	0.93 (0.82–1.05)	0.96 (0.85–1.09)
Sickness absence (reference 0 gross days)				
1–90 gross days	1.26 (0.98–1.63)	1.28 (0.99–1.64)	1.05 (0.96–1.15)	1.05 (0.95–1.16)
>90 gross days	0.78 (0.52–1.17)	0.78 (0.51–1.19)	0.95 (0.84–1.06)	0.98 (0.87–1.10)
Marital status (reference unmarried)				
Married	0.87 (0.71–1.06)	0.85 (0.68–1.06)	0.90 (0.78–1.03)	0.90 (0.78–1.04)
Divorced	0.83 (0.63–1.11)	0.84 (0.63–1.13)	0.72 (0.53–0.97)	0.75 (0.56–1.02)
Disability pension	1.26 (1.02–1.57)	1.18 (0.93–1.48)	1.29 (1.21–1.37)	1.30 (1.21–1.40)
Region of birth (reference former Yugoslavia)				
Afghanistan	0.54 (0.35–0.83)	0.49 (0.32–0.76)	Not applicable	Not applicable
Iraq	0.75 (0.61–0.94)	0.77 (0.62–0.97)		
Iran	0.88 (0.66–1.16)	0.86 (0.64–1.14)		
Other Middle East	0.77 (0.58–1.01)	0.78 (0.59–1.03)		
Somalia	0.83 (0.67–1.04)	0.89 (0.71–1.12)		
Other Africa	0.87 (0.67–1.12)	0.83 (0.64–1.06)		
Other Europe	0.94 (0.71–1.24)	0.93 (0.70–1.23)		
The Americas and Asia	0.93 (0.69–1.23)	0.94 (0.71–1.24)		
Comorbidities				
Cancer	1.59 (1.12–2.24)	1.50 (1.06–2.12)	0.99 (0.84–1.16)	0.97 (0.83–1.14)
Attention-deficit hyperactivity disorder	0.94 (0.63–1.40)	0.94 (0.63–1.42)	0.99 (0.91–1.08)	1.01 (0.92–1.10)
Substance use disorder	0.95 (0.78–1.16)	1.07 (0.87–1.31)	0.80 (0.74–0.86)	0.83 (0.77–0.90)
Bipolar disorder	1.01 (0.60–1.70)	0.81 (0.49–1.34)	1.08 (0.94–1.24)	0.99 (0.86–1.15)
Personality disorder	1.24 (0.86–1.79)	1.35 (0.91–2.00)	0.88 (0.76–1.02)	0.87 (0.75–0.99)
Common mental disorder	1.10 (0.96–1.27)	1.04 (0.91–1.20)	1.02 (0.97–1.06)	1.01 (0.96–1.06)
Cardiovascular disease	0.74 (0.41–1.34)	0.79 (0.43–1.45)	0.93 (0.76–1.14)	0.96 (0.78–1.17)
Musculoskeletal disease	0.77 (0.58–1.01)	0.78 (0.59–1.03)	0.81 (0.73–0.91)	0.83 (0.74–0.92)
Previous suicide attempt	0.78 (0.53–1.15)	0.74 (0.50–1.10)	0.86 (0.77–0.97)	0.91 (0.81–1.03)
Previous medication use				
Antidepressant drugs	1.30 (1.09–1.56)	1.33 (1.08–1.63)	1.08 (1.01–1.15)	1.01 (0.95–1.09)
Anxiolytic/hypnotic drugs	1.08 (0.97–1.21)	1.01 (0.89–1.15)	1.11 (1.07–1.14)	1.11 (1.07–1.15)
Mood stabilisers	1.36 (0.92–2.01)	1.37 (0.92–2.04)	1.19 (1.04–1.35)	1.11 (0.97–1.27)
Type of diagnosis (reference other F21/22/24/28)				
Schizophrenia/schizoaffective disorder (F20/F25)	1.66 (1.22–2.24)	1.72 (1.27–2.34)	1.37 (1.22–1.54)	1.32 (1.17–1.48)
Acute/ transient psychosis (F23)	1.14 (0.86–1.51)	1.17 (0.89–1.55)	1.06 (0.96–1.17)	1.09 (0.98–1.20)
Unspecified (F29)	1.23 (0.94–1.62)	1.31 (1.00–1.73)	1.30 (1.18–1.43)	1.33 (1.21–1.47)

a. Adjusted for all factors shown.

b. Indicates statistically significant associations.

### Initiation of LAI or clozapine use

A larger proportion of the Swedish-born individuals initiated clozapine use (4.7% of the Swedish-born *v*. 3.3% of the refugees during mean follow-up of 3.7 years, s.d. 1.7), whereas the reverse was true for LAI use (13.2% *v*. 18.3%, mean follow-up time 3.5 years, s.d. 1.8; Supplementary Table 4). During the follow-up, 17.1% of the refugees were diagnosed with schizophrenia, and the corresponding proportion for Swedish-born persons was 14.2%. In the adjusted Cox regression analyses (Table 3), refugees were almost 50% more likely to initiate LAIs compared with Swedish-born persons (adjusted hazard ratio 1.49, 95% CI 1.30–1.71). The countries of origin that were significantly associated with LAI initiation were Iraq, Iran, ‘other Africa’ and ‘other Europe’, compared with former Yugoslavia. Female gender was associated with decreased hazard of initiation compared with male gender (adjusted hazard ratio 0.74, 95% CI 0.66–0.83), whereas married persons (compared with unmarried) and those living in towns and suburbs (compared with dense cities) were less likely to initiate LAIs. Being unemployed and receiving disability pension were associated with increased likelihood of earlier initiation.

**Table 3:** Unadjusted and adjusted hazard ratios displaying the time to long-acting injectable drug initiation for both refugees and Swedish-born individuals (*N* = 10 537)

	Unadjusted hazard ratio (95% CI)	Adjusted hazard ratio (95% CI)
Refugee status (reference Swedish-born)		
Refugee	1.47 (1.30–1.67)^a^	1.49 (1.30–1.71)^a^
Gender (reference male)	0.69 (0.62–0.78)^a^	0.74 (0.66–0.83)^a^
Age, years (reference 18–23 years)		
24–29	1.01 (0.91–1.15)	0.99 (0.87–1.12)
30–35	0.95 (0.83–1.09)	1.00 (0.87–1.16)
Education level (reference low <10 years)		
Medium (10–12 years	1.15 (0.78–1.13)	0.90 (0.74–1.09)
High (>12 years)	0.97 (0.87–1.08)	1.06 (0.94–1.19)
Living area (reference dense cities)		
Towns and suburbs	0.77 (0.69–0.86)^a^	0.78 (0.70–0.88)^a^
Rural areas	0.88 (0.76–1.02)	0.92 (0.80–1.07)
Marital status (reference unmarried)		
Married	0.56 (0.42–0.74)^a^	0.53 (0.39–0.70)^a^
Divorced	1.03 (0.73–1.44)	0.91 (0.64–1.29)
Unemployment (reference no unemployment)		
1–180 days	1.29 (1.15–1.45)^a^	1.26 (1.12–1.42)
>180 days	1.38 (1.14–1.68)^a^	1.33 (1.09–1.62)^a^
Sickness absence (reference 0 days)		
1–90 gross days	0.85 (0.69–1.04)	1.15 (0.77–1.18)
>90 gross days	0.80 (0.63–1.02)	0.88 (0.69–1.13)
Disability pension	1.26 (1.09–1.45)^a^	1.38 (1.19–1.60)^a^
Time in Sweden (reference 3–5 years)		
6–10 years	1.17 (0.82–1.68)	
>10 years	0.99 (0.73–1.35)	
Region of birth (reference Swedish-born)		
Afghanistan	1.57 (0.94–2.61)	
Iraq	1.62 (1.25–2.10)^a^	
Iran	1.75 (1.19–2.59)^a^	
Other middle east	1.18 (0.77–1.80)	
Somalia	1.24 (0.92–1.69)	
Other Africa	2.15 (1.59–2.90)^a^	
Former Yugoslavia	1.15 (0.86–1.55)	
Other Europe	1.65 (1.07–2.54)^a^	
The Americas and Asia	1.42 (0.91–2.21)	

a. Indicates statistically significant associations.

Refugees were 28% less likely to initiate clozapine compared with Swedish-born individuals in the crude analysis (hazard ratio 0.72, 95% CI 0.54–0.95). When controlling for sociodemographic factors, the association was no longer statistically significant, nor were the associations of any of the countries of birth (Table 4). Similar to LAI initiation, women were 30% less likely to initiate clozapine compared with men (adjusted hazard ratio 0.70, 95% CI 0.57–0.87). In the adjusted models, the likelihood of initiating clozapine was lower among older study participants and those who were married (compared with unmarried), but higher among those who received disability pension before cohort entry (adjusted hazard ratio 1.66, 95% CI 1.30–2.11).

**Table 4:** Unadjusted and adjusted hazard ratios displaying the time to clozapine initiation for both refugees and Swedish-born individuals (*N* = 10 555)

	Unadjusted hazard ratio (95% CI)	Adjusted hazard ratio (95% CI)
Refugee status (reference Swedish-born)		
Refugee	0.72 (0.54–0.95)^a^	0.84 (0.63–1.13)
Gender (reference male)	0.68 (0.56–0.84)^a^	0.70 (0.57–0.87)^a^
Age (reference 18–23 years)		
24–29 years	0.69 (0.56–0.85)^a^	0.65 (0.53–0.81)^a^
30–35 years	0.50 (0.39–0.64)^a^	0.53 (0.40–0.69)^a^
Educational level (reference low <10 years)		
Medium (10–12 years)	0.72 (0.50–1.03)	0.89 (0.61–1.28)
High (>12 years)	0.95 (0.79–1.15)	1.20 (0.97–1.47)
Living area (reference dense cities)		
Towns and suburbs	1.02 (0.84–1.24)	0.99 (0.81–1.20)
Rural areas	0.98 (0.76–1.27)	0.93 (0.72–1.21)
Marital status (reference unmarried)		
Married	0.13 (0.05–0.35)^a^	0.19 (0.07–0.51)^a^
Divorced	0.35 (0.13–0.94)^a^	0.56 (0.20–1.51)
Unemployment (reference no unemployment)		
1–180 days	0.88 (0.70–1.09)	0.87 (0.70–1.09)
>180 days	1.09 (0.77–1.55)	1.31 (0.91–1.87)
Sickness absence (reference 0 days)		
1–90 gross days	0.77 (0.53–1.13)	0.95 (0.65–1.41)
>90 gross days	0.64 (0.40–1.03)	0.86 (0.53–1.40)
Disability pension	1.53 (1.21–1.93)^a^	1.66 (1.30–2.11)^a^
Time in Sweden (reference 3–5 years)		
6–10 years	1.37 (0.56–3.34)	
>10 years	1.26 (0.59–2.73)	
Region of birth (reference Swedish-born)		
Afghanistan	0.55 (0.14–2.22)	
Iraq	0.74 (0.39–1.38)	
Iran	0.87 (0.36–2.10)	
Other Middle East	0.74 (0.31–1.78)	
Somalia	0.70 (0.36–1.36)	
Other Africa	0.63 (0.26–1.52)	
Former Yugoslavia	0.91 (0.52–1.57)	
Other Europe	0.41 (0.10–1.62)	
The Americas and Asia	0.56 (0.18–1.73)	

a. Indicates statistically significant associations.

## Discussion

The results of this nationwide study suggest that being a refugee is associated with a lower prevalence of antipsychotic use compared with Swedish-born persons during the first year after diagnosis of non-affective psychotic disorder. However, these differences became attenuated over time, and at 5 years after first diagnosis, the prevalence of antipsychotic use was similar among refugees and their Swedish-born peers. Among refugees, having a high educational level (>12 years), receiving disability pension, previous use of antidepressants and having a diagnosis of schizophrenia/schizoaffective disorder were positively associated with antipsychotic use at 1 year after first diagnosis. Refugees who had received unemployment benefits, as well as those born in Afghanistan and Iraq (compared with those born in former Yugoslavia), had decreased risk of using antipsychotics. The Cox proportional regression analyses implied that refugees were 50% more likely to initiate LAIs compared with Swedish-born individuals, but were 28% less likely to commence clozapine.

The results of the present study are partly aligned with those of a Finnish study which observed that migrants were less likely to use antipsychotic medication compared with the general population.^15^ The relatively low prevalence rate of antipsychotic use at 1 year observed in our study (37.1% in refugees and 42.2% in Swedish-born persons) is not surprising as the cohort also included acute and transient psychotic illness, which may not require long-term antipsychotic use, and is in line with the previous study reporting that 60.5% of individuals with psychosis did not initiate or discontinued antipsychotic use during the first year. Although a similar pattern could be observed for the refugees in this study during the first year after diagnosis, this was not the case after 5 years; indeed, the proportion of those receiving antipsychotics at the 5-year follow-up was similar in refugees and Swedish-born individuals. One possible explanation is that refugees might not seek help until a later stage of their illness than Swedish-born individuals, perhaps because of a mistrust or lack of knowledge about Swedish healthcare systems, language barriers (which could limit the ability to access healthcare) or perceived stigma related to their illness and/or medication use.^15^ Over time, refugees may gain trust in institutions and an increased understanding of the healthcare system, which may partly explain the rising pattern of antipsychotic use that was observed for refugees over the 5 years of follow-up. Alternatively, refugees may have more chronic course of illness, necessitating antipsychotic use. Swedish-born individuals may have had milder or less chronic illness course compared with refugees, and not require longer-term treatment to the same extent.^24^ In support of this hypothesis, we observed that refugees were more likely to be diagnosed with schizophrenia during the 5-year follow-up than their Swedish-born peers. Previous research has highlighted that there are often delays until a formal diagnosis of schizophrenia is established, which is often because of miscommunication with clinicians. This could entail an underestimation of schizophrenia diagnoses in the register-based data, which could partly be related to stigma associated with this diagnosis.^25^ It is not known to what extent this differs between refugees and individuals born in the host country.

Previous research has shown that non-use of antipsychotics in schizophrenia is associated with younger age, low socioeconomic status, poor insight, substance use disorders, negative attitudes toward medication, belonging to an ethnic minority and experiencing barriers to healthcare.^6^ The possible role of being a refugee has not been investigated before, and no studies have been conducted to identify these factors specifically among refugees. In the present study, having attained a high educational level was positively associated with antipsychotic use at 1 year after diagnosis in both refugees and Swedish-born individuals. Among refugees, education could act as a marker for more established networks and healthcare navigation skills. Higher education can also be considered as a marker of better language skills. Having previously used antidepressants was also associated with increased chances of using antipsychotics at 1 year after diagnosis, which might suggest that those who had previously used antidepressants had a more positive overall attitude toward medication in general. It is also possible that individuals who had previously used antidepressants were more accustomed to clinical procedures through having regular contact with healthcare, something that previous research has found to be associated with favourable prognosis among individuals with non-affective psychosis.^26^

We additionally observed that disability pension was associated with increased chances of antipsychotic use. In Sweden, disability pension is granted to individuals aged 19–64 years who, because of disease or injury, have a reduced work capacity;^27^ thus, receipt of disability pension indicates the presence of a severe illness. This is in line with the finding that having been diagnosed with schizophrenia or schizoaffective disorder at baseline was associated with a higher chance of antipsychotic use, compared with other more acute or possibly transient forms of psychotic disorders.

Similar to previous research that found that migrants from the Middle East were less likely to use antipsychotics,^15^ this study also found that refugees born in Afghanistan and Iraq were less likely to use antipsychotics than those born in former Yugoslavia at 1 year after first diagnosis. These findings may be a result of cultural differences in how mental disorders and their pharmacotherapies are perceived and/or differences in health behaviours. The same previous study found that migrants who had recently arrived in the host country were less likely to use antipsychotics; however, the present study could not replicate these findings.

Our finding that refugees were more likely to initiate LAIs compared with Swedish-born individuals might suggest that refugees were more unwell (e.g. experienced relapse of psychosis) or that they were perceived to be less likely to adhere to their medication regimen. Clozapine initiation, on the other hand, was less common for refugees compared with Swedish-born persons. This is somewhat concerning as refugees were also more likely to be diagnosed with schizophrenia (a disorder for which antipsychotic maintenance treatment is particularly indicated). A previous systematic review also found a pattern of underutilisation of clozapine in ethnic minorities, which was largely explained by systematic ethnic disparities in terms of clozapine practice.^28^ Because of the severe health risks associated with use, clozapine necessitates regular and mandatory blood monitoring, which can be burdensome and requires the patient to be vigilant of these risk (e.g. being aware of the first signs of agranulocytosis). It is therefore likely that clinicians may be less willing to prescribe clozapine to refugees, who may be perceived to be unable to understand these risks (because of language barriers) or unlikely to be able to comply with regular laboratory visits.^15,28,29^ As such, refugees may be more likely to be prescribed LAIs as an alternative to clozapine.

The main strength of this study is its use of large, high-quality nationwide registers, providing access to multiple sociodemographic and clinical factors, medication and outcome measures. Moreover, a long inclusion window guaranteed sufficiently statistical power for the main analyses, and the use of register-based data resulted in practically no loss to follow-up. We utilised the validated PRE2DUP method for constructing drug use periods, which were the bases for defining point prevalence of use. We measured 2-week point prevalence, which represents a more accurate estimate of the current use status than period prevalence (e.g. over 6 months). Point prevalence is more consistent with estimates of use derived in clinical studies where patients are asked whether they are currently using medication. The results are generalisable to countries with healthcare systems resembling that of Sweden (e.g. most other European countries, Australia and Canada). Nevertheless, this study has limitations. Study inclusion criteria required persons to have been residing in Sweden for at least 3 years before the first diagnosis, to ensure that we captured those treated for the first time (i.e. incident cases). However, it also means that our study lacks persons who have very recently entered the country and who likely face even greater challenges accessing mental health services. We also lacked data from primary care visits and therefore cannot know if persons were treated for psychosis in primary care before their first contact in specialised care. For this reason, we allowed initiations of antipsychotics during 3 months before the first diagnosis and avoided excluding these persons. Although several crucial covariates such as gender, age and educational level were controlled for, there can be a risk of residual confounding owing to personal characteristics that were not adjusted for, such as alcohol consumption, illicit drug use, language skills, experience of stigma and family history of psychotic disorders.^30^ Moreover, we were only able to adjust for treatment for comorbid conditions in specialised healthcare and are therefore missing those with milder health problems treated in primary care. Missing data on educational level could possibly have underestimated the true number of participants with medium and high educational level. Nevertheless, the proportions of missing values for education was similar for both refugees and Swedish-born individuals, which makes this potential misclassification non-differential. This study focused on refugees specifically, and future research should aim to compare refugees with non-refugee migrants.

In conclusion, we observed that refugees were less likely to use antipsychotic drugs during the first 2 years after first treatment for first-episode non-affective psychosis compared with their Swedish-born peers. However, there were no significant differences when 5 years had passed, which could suggest differences in the course of illness or that refugees had gained more health literacy and their trust in Swedish healthcare had been enhanced over time. These findings suggest that young refugees with non-affective psychosis may need specifically targeted interventions to enhance their likelihood of using antipsychotics at an earlier stage of their illness, as this could potentially improve their disease prognosis. Factors such as previous unemployment, low educational level, previous suicide attempts, comorbid substance use disorder and being born in Afghanistan and Iraq were associated with decreased chances of antipsychotic use. Special focus should therefore be directed to these groups, to improve initiation and adherence with medication regimens.

## Data Availability

The data that support the findings of this study are available from the Swedish government agencies, but restrictions apply to the availability of these data, which were used under license for the current study and so are not publicly available.

## Supplementary material

For supplementary material accompanying this paper visit http://doi.org/10.1192/bjo.2023.38. click here to view supplementary material

## References

[1] Kahn RS, Sommer IE, Murray RM, Meyer-Lindenberg A, Weinberger DR, Cannon TD, Schizophrenia. Nat Rev Dis Prim 2015; 1: 150677. 10.1038/nrdp.2015.67 27189524

[2] Schneider-Thoma J, Chalkou K, Dörries C, Bighelli I, Ceraso A, Huhn M, Comparative efficacy and tolerability of 32 oral and long-acting injectable antipsychotics for the maintenance treatment of adults with schizophrenia: a systematic review and network meta-analysis. Lancet 2022; 399: 824–36. 35219395 10.1016/S0140-6736(21)01997-8

[3] Socialstyrelsen. Nationella riktlinjer för antipsykotisk läkemedelsbehandling vid Schizofreni och Schizofreniliknande tillstånd. [National guidelines for antipsychotic drug treatment in schizophrenia and schizophrenia-like conditions.] Artik, 2014 (https://www.socialstyrelsen.se/globalassets/sharepoint-dokument/artikelkatalog/nationella-riktlinjer/2016-6-7.pdf).

[4] Haddad P, Brain C, Scott J. Nonadherence with antipsychotic medication in schizophrenia: challenges and management strategies. Patient Relat Outcome Meas 2014; 5: 43–62. 25061342 10.2147/PROM.S42735 PMC4085309

[5] Velligan DI, Sajatovic M, Hatch A, Kramata P, Docherty JP. Why do psychiatric patients stop antipsychotic medication? A systematic review of reasons for nonadherence to medication in patients with serious mental illness. Patient Prefer Adherence 2017; 11: 449–68. 28424542 10.2147/PPA.S124658 PMC5344423

[6] García S, Martínez-Cengotitabengoa M, López-Zurbano S, Zorrilla I, López P, Vieta E, Adherence to antipsychotic medication in bipolar disorder and schizophrenic patients. J Clin Psychopharmacol 2016; 36: 355–71. 27307187 10.1097/JCP.0000000000000523 PMC4932152

[7] Greene M, Yan T, Chang E, Hartry A, Touya M, Broder MS. Medication adherence and discontinuation of long-acting injectable versus oral antipsychotics in patients with schizophrenia or bipolar disorder. J Med Econ 2018; 21: 127–34. 28895758 10.1080/13696998.2017.1379412

[8] Tiihonen J, Mittendorfer-Rutz E, Majak M, Mehtälä J, Hoti F, Jedenius E, Real-world effectiveness of antipsychotic treatments in a nationwide cohort of 29 823 patients with schizophrenia. JAMA Psychiatry 2017; 74: 686–93. 28593216 10.1001/jamapsychiatry.2017.1322 PMC5710250

[9] Taipale H, Tanskanen A, Mehtälä J, Vattulainen P, Correll CU, Tiihonen J. 20-year follow-up study of physical morbidity and mortality in relationship to antipsychotic treatment in a nationwide cohort of 62,250 patients with schizophrenia (FIN20). World Psychiatry 2020; 19: 61–8. 31922669 10.1002/wps.20699 PMC6953552

[10] Correll CU, Solmi M, Croatto G, Schneider LK, Rohani-Montez SC, Fairley L, Mortality in people with schizophrenia: a systematic review and meta-analysis of relative risk and aggravating or attenuating factors. World Psychiatry 2022; 21: 248–71. 35524619 10.1002/wps.20994 PMC9077617

[11] Hollander AC, Dal H, Lewis G, Magnusson C, Kirkbride JB, Dalman C. Refugee migration and risk of schizophrenia and other non-affective psychoses: cohort study of 1.3 million people in Sweden. BMJ 2016; 352: i1030. 10.1136/bmj.i1030 PMC4793153 26979256

[12] Reed RV, Fazel M, Jones L, Panter-Brick C, Stein A. Mental health of displaced and refugee children resettled in low-income and middle-income countries: risk and protective factors. Lancet 2012; 379: 250–65. 21835460 10.1016/S0140-6736(11)60050-0

[13] Björkenstam E, Helgesson M, Norredam M, Sijbrandij M, de Montgomery CJ, Mittendorfer-Rutz E. Differences in psychiatric care utilization between refugees, non-refugee migrants and Swedish-born youth. Psychol Med 2022; 52(7): 1365–75. 32914741 10.1017/S0033291720003190

[14] Brendler-Lindqvist M, Norredam M, Hjern A. Duration of residence and psychotropic drug use in recently settled refugees in Sweden - a register-based study. Int J Equity Health 2014; 13: 122. 25526935 10.1186/s12939-014-0122-2 PMC4297375

[15] Lehti V, Taipale H, Gissler M, Tanskanen A, Elonheimo M, Tiihonen J, Continuity of antipsychotic medication use among migrant and Finnish-born populations with a psychotic disorder: a register-based study. Psychol Med 2023; 53(3): 833–43. 10.1017/S003329172100218X 34074352

[16] Lehti V, Suvisaari J, Gissler M, Markkula N. Purchases of psychotropic drugs among the migrant population in Finland: a nationwide register-based cohort study. Eur J Public Health 2020: 30(6): 1152–7. 32754762 10.1093/eurpub/ckaa117

[17] Samkange-Zeeb F, Samerski S, Doos L, Humphris R, Padilla B, Bradby H. “It's the first barrier” – lack of common language a major obstacle when accessing/providing healthcare services across Europe. Front Sociol 2020; 5: 557563. 33869495 10.3389/fsoc.2020.557563 PMC8022480

[18] Krendl AC, Pescosolido BA. Countries and cultural differences in the stigma of mental illness: the East–West Divide. J Cross Cult Psychol 2020; 51: 149–67.

[19] Bosqui T, Väänänen A, Koskinen A, Buscariolli A, O'reilly D, Airila A, Antipsychotic medication use among working-age first-generation migrants resident in Finland: an administrative data linkage study. Scand J Public Health 2020; 48(1): 64–71. 30973081 10.1177/1403494819841960

[20] Kessler RC, Amminger GP, Aguilar-Gaxiola S, Alonso J, Lee S, Üstün TB. Age of onset of mental disorders: a review of recent literature. Curr Opin Psychiatry 2007; 20: 359–64. 17551351 10.1097/YCO.0b013e32816ebc8c PMC1925038

[21] Tanskanen A, Taipale H, Koponen M, Tolppanen A-M, Hartikainen S, Ahonen R, From prescription drug purchases to drug use periods – a second generation method (PRE2DUP). BMC Med Inform Decis Mak 2015; 15: 21. 25890003 10.1186/s12911-015-0140-z PMC4382934

[22] Zou G. A modified Poisson regression approach to prospective studies with binary data. Am J Epidemiol 2004; 159: 702–6. 15033648 10.1093/aje/kwh090

[23] Pedersen AB, Mikkelsen EM, Cronin-Fenton D, Kristensen NR, Pham TM, Pedersen L, Missing data and multiple imputation in clinical epidemiological research. Clin Epidemiol 2017; 9: 157–66. 28352203 10.2147/CLEP.S129785 PMC5358992

[24] Brandt L, Henssler J, Müller M, Wall S, Gabel D, Heinz A. Risk of psychosis among refugees: a systematic review and meta-analysis. JAMA Psychiatry 2019; 76: 1133–40. 31411649 10.1001/jamapsychiatry.2019.1937 PMC6694397

[25] Patel P, Bernays S, Dolan H, Muscat DM, Trevena L. Communication experiences in primary healthcare with refugees and asylum seekers: a literature review and narrative synthesis. Int J Environ Res Public Health 2021; 18: 1469. 33557234 10.3390/ijerph18041469 PMC7913992

[26] Correll CU, Kim E, Sliwa JK, Hamm W, Gopal S, Mathews M, Pharmacokinetic characteristics of long-acting injectable antipsychotics for schizophrenia: an overview. CNS Drugs 2021; 35: 39–59. 33507525 10.1007/s40263-020-00779-5 PMC7873121

[27] Försäkringskassan. *Sjukersättning*. [Sickness benefits.] Försäkringskassan, 2023 (https://www.forsakringskassan.se/privatpers/sjuk/sjuk_minst_1_ar/sjukersattning).

[28] Williams JC, Harowitz J, Glover J, Tek C, Srihari V. Systematic review of racial disparities in clozapine prescribing. Schizophr Res 2020; 224: 11–8. 33183948 10.1016/j.schres.2020.07.023

[29] Nucifora FC, Mihaljevic M, Lee BJ, Sawa A. Clozapine as a model for antipsychotic development. Neurotherapeutics 2017; 14: 750–61. 28653280 10.1007/s13311-017-0552-9 PMC5509641

[30] Laursen TM, Munk-Olsen T, Vestergaard M. Life expectancy and cardiovascular mortality in persons with schizophrenia. Curr Opin Psychiatry 2012; 25: 83–8. 22249081 10.1097/YCO.0b013e32835035ca

