# Supplementary material for: Antipsychotic treatment patterns in refugees and their Swedish-born peers with first-episode non-affective psychosis: findings from the REMAIN study
Source: BJPsych Open. 2023 Apr 4;9(3):e57. doi: 10.1192/bjo.2023.38 (PMC10134221; doi:10.1192/bjo.2023.38)
Supplement: Supplementary file 1 [file S2056472423000388sup001.docx]

# Supplementary material

**Supplementary Table 1.** All covariates included in this study,

| **Covariates** | **ICD10¹ / ATC-code²** | **Definition** | **Time of measurement** |
| --- | --- | --- | --- |
| **Sociodemographic covariates** |  |  |  |
| Age at first diagnosis (years) | N/A | 18-23, 24-29, 30-35 | The day of first diagnosis |
| Sex | N/A | Male, female | The day of first diagnosis |
| Educational level (years) | N/A | <10, 10-12, >12 | December 31? |
| Living area | N/A | Cities, towns, rural | December 31 the year prior to first diagnosis |
| Marital status | N/A | Unmarried, married, divorced | December 31 the year prior to first diagnosis |
| Unemployment (days) | N/A | 0, 1-180, >180 | Gross days during the 360-1 days prior to first diagnosis |
| Sickness Absence (days) | N/A | 0, 1-90, >90 | Gross days during the 360-1 days prior to first diagnosis |
| Disability pension | N/A | No, Yes | The day prior to first diagnosis |
| Region of birth* | N/A | [Appendix A] | The day of first diagnosis |
| Age at immigration (years)* | N/A | 0-31 | The day of first diagnosis |
| Duration of formal residency in Sweden (years)* | N/A | 3-5, 6-10, >10 | The day of first diagnosis |
| **Clinical covariates** |  |  |  |
| Substance use disorders (SUD) | ICD10: F10-F19 | No, Yes | Three years prior to first diagnosis |
| Attention-deficit hyperactivity disorder (ADHD) | ICD10: F90 | No, Yes | Three years prior to first diagnosis |
| Bipolar disorder | ICD10: F30-F31 | No, Yes | Three years prior to first diagnosis |
| Personality disorder | ICD10: F60-F69 | No, Yes | Three years prior to first diagnosis |
| Depression | ICD10: F32-F33 | No, Yes | Three years prior to first diagnosis |
| Anxiety | ICD10: F40-F43 | No, Yes | Three years prior to first diagnosis |
| Musculoskeletal disease | ICD10: M00-M99 | No, Yes | Three years prior to first diagnosis |
| Cardiovascular disease | ICD10: 100-199 | No, Yes | Three years prior to first diagnosis |
| Cancer | ICD10: C00-C99 | No, Yes | Three years prior to first diagnosis |
| Type of psychotic disorder | ICD10: F20-F29 | No, Yes | The day of first diagnosis |
| Previous suicide attempt | ICD10: X60-X84, Y10-Y34 | No, Yes | Three years prior to first diagnosis |
| **Previous medication use covariates** |  |  |  |
| Antidepressants | ATC: N06A | No, Yes | Three months prior to first diagnosis |
| Anxiolytic drugs | ATC: N05B | No, Yes | Three months prior to first diagnosis |
| Hypnotic drugs | ATC: N05C | No, Yes | Three months prior to first diagnosis |
| Mood stabilisers | ATC: N03AF01, N03AG01, N03AX09, N05AN01 | No, Yes | Three months prior to first diagnosis |

*Only applies to refugees
¹ ICD-10: International Classification of Diseases version 10
² ATC: Anatomic Therapeutic Chemical Classification system

**Supplementary Table 2.** Description of the categorisation of birth countries used throughout the study.

| **Country Categories** | **N (%)** | **Description** |
| --- | --- | --- |
| Afghanistan | 86 (5.2) |  |
| Iraq | 309 (18.7) |  |
| Iran | 121 (7.3) |  |
| Somalia | 294 (17.8) |  |
| Former Yugoslavia | 292 (17.6) | Bosnia-Hercegovina, Yugoslavia, Croatia, Macedonia, Serbia, Serbia and Montenegro, Slovenia |
| Other Africa | 185 (11.2) | Algeria, Angola, Arab republic Egypt, Burundi, Cameroon, Central African Republic, Djibouti, Egypt, Eritrea, Ethiopia, Gambia, Ghana, Guinea, Ivory Coast, Kenya, Kongo, Kongo Democratic Republic, Liberia, Libya, Mali, Morocco, Nigeria, Rwanda, Senegal, Sierra Leone, Sudan, South Africa, Tanzania, Togo, Uganda |
| Other Europe | 103 (6.2) | Albania, Armenia, Azerbaijan, Belgium, Bulgaria, Cyprus, Denmark, Georgia, Italy, Kosovo, Latvia, Poland, Romania, Russia, Slovakia, Soviet Union, Chez republic, Germany, Ukraine, Hungary, Belarus. |
| Other Middle East | 154 (9.3) | United Arab Emirate, Bahrain, Gaza area, Israel, Jordan, Kuwait, Lebanon, Palestine, Saudi Arabia, Syria, Turkey, Yemen |
| Asia | 54 (3.3) | Bangladesh, Philippines, India, Kazakhstan, Kina, Kirgizstan, Laos, Myanmar, Nepal, Pakistan, Sri Lanka, Thailand, Turkmenistan, Uzbekistan, Vietnam, Vietnam rep |
| The Americas | 58 (3.5) | Chile, Colombia, Ecuador, El Salvador, Guatemala, Mexico, Peru |

**Supplementary Table 3.** Point prevalence (%) of antipsychotic drug use, measured every six months for a maximum of five years after diagnosis (t1-t10) for both refugees and Swedish-born.

| **Time Point (years)** | **Refugees** | **95% CI** | **Swedish-born** | **95% CI** |
| --- | --- | --- | --- | --- |
| t1 (0.5) | 40.08 | 37.60 - 42.61 | 46.93 | 45.84 - 48.02 |
| t2 (1) | 37.11 | 34.59 - 39.70 | 42.18 | 41.08 - 43.29 |
| t3 (1.5) | 35.85 | 33.27 - 38.51 | 40.30 | 39.18 - 41.43 |
| t4 (2) | 38.42 | 35.70 - 41.20 | 39.59 | 38.45 - 40.75 |
| t5 (2.5) | 39.86 | 37.02 - 42.77 | 39.09 | 37.92 - 40.27 |
| t6 (3) | 39.53 | 36.58 - 42.56 | 39.97 | 38.76 - 41.20 |
| t7 (3.5) | 40.52 | 37.56 - 43.64 | 39.70 | 38.45 - 40.96 |
| t8 (4) | 39.47 | 36.33 - 42.70 | 39.82 | 38.53 - 41.12 |
| t9 (4.5) | 39.93 | 36.67 - 43.28 | 40.58 | 39.25 - 41.93 |
| t10 (5) | 41.09 | 37.70 - 44.58 | 40.35 | 38.97 - 41.75 |

**Supplementary Table 4.** Proportions of the study participants who initiated long-acting injectables (LAI) or clozapine use or were diagnosed with schizophrenia during the follow-up period*,* comparing refugees and Swedish-born persons with first-episode non-affective psychosis.

|  | **Refugees** N=1656 (15.7%) |  | **Swedish-born** N=8908 (84.3%) | **Total** N=10,564 (100%) |
| --- | --- | --- | --- | --- |
| **Clozapine** | 55 (3.3) |  | 419 (4.7) | 474 (4.5) |
| **LAI** | 303 (18.3) |  | 1171 (13.2) | 1474 (14.0) |
| **Schizophrenia (F20)** | 283 (17.1) |  | 1267 (14.2) | 1550 (14.7) |
